# Detection of circulating immune complexes in feline leishmaniosis: first evidence and diagnostic implications

**DOI:** 10.1007/s11259-026-11120-8

**Published:** 2026-03-02

**Authors:** Ana González, Nuria Parody, Ana Renshaw, Gemma Navarro, Leticia Montañés, María Eugenia Lebrero, Sergio Villanueva-Saz, Roser Fisa, Diana Marteles-Aragüés, Xavier Roca-Geronès

**Affiliations:** 1 Clinical Immunology Laboratory, Veterinary Faculty, University of Zaragoza, Calle Miguel Servet, 177, Zaragoza, 50013 Spain; 2 Department of Animal Pathology, Veterinary Faculty, University of Zaragoza, Calle Miguel Servet, 177, Zaragoza, 50013 Spain; 3 R&D Unit Allergy and Immunology, LETI Pharma S.L.U, Calle del Sol, 5, Tres Cantos, Madrid, 28760 Spain; 4 Centro de Biología Molecular Severo Ochoa, CSIC-Universidad Autónoma de Madrid, Cantoblanco, Madrid, Spain; 5 Centro Veterinario Husse, Calle Guara, 15, Cuarte de Huerva, Zaragoza, 50410 Spain; 6 Instituto Agroalimentario de Aragón-IA2 (Universidad de Zaragoza-CITA), Calle Miguel Servet, 177, Zaragoza, 50013 Spain; 7 Departament de Biologia, Salut I Medi Ambient, Facultat de Farmacia, Universitat de Barcelona, Av. De Joan XXIII, Barcelona, Barcelona, 08028 Spain

**Keywords:** Diagnostic, Feline leishmaniosis, Immune complexes, Leishmania infantum, PEG-ELISA, Spain

## Abstract

Feline leishmaniosis caused by *Leishmania infantum* is a zoonotic, vector-borne disease. While dogs are the main reservoir and exhibit immune-response–dependent spectra of illness, cats most often show lymphadenomegaly, dermatologic lesions, ocular or oral disease, weakness, weight loss, and clinicopathologic changes such as non-regenerative anemia, hyperglobulinemia, and proteinuria. Diagnosis benefits from combining serology and molecular tests, since single methods have limited clinical relevance. Circulating immune complexes drive many lesions in canine leishmaniosis and correlate with severity; a feline, *Leishmania*-specific circulating immune complexes assay has been lacking. This report describes an eight-year-old spayed European Shorthair cat from Spain with unilateral uveitis as the main clinical manifestation associated with *Leishmania infantum* infection. *Leishmania* infection was confirmed by positive immunochromatographic rapid test, high anti-*Leishmania* enzyme-linked immunosorbent assay and Western Blot reactivity, molecular test on blood and aqueous humor, and parasite culture with typing as *L. infantum*. A *Leishmania*-specific enzyme-linked immunosorbent assay adapted for detecting feline circulating immune complexes was developed and set up and the level of this promising biomarker was measured longitudinally during short-term follow-up. The cat received allopurinol (20 mg/kg every 24 h) plus topical anti-inflammatories. Clinical uveitis resolved within thirty days with concurrent improvements in activity and ocular findings. The kinetics of circulating immune complexes paralleled those of anti-*Leishmania* antibodies measured by enzyme-linked immunosorbent assay. This is the first clinical application of circulating immune complexes measure in feline leishmaniosis. The isolation and detection of circulating immune complexes appear to be a useful biomarker for diagnosis, staging, and monitoring treatment response in cats.

## Background

Feline leishmaniosis (FeL) is a zoonotic, vector-borne disease caused by *Leishmania infantum*. Dogs are regarded as the principal vertebrate reservoir for human infection (Alvar et al. 2004). In dogs, the disease presents with a wide spectrum of clinical manifestations, from subclinical infection to severe and potentially fatal illness, largely influenced by variations in the host’s cell-mediated immune response and genetic background (Hosein et al. 2017).

In cats, the most common clinical manifestations of this disease include peripheral lymphadenomegaly, cutaneous and mucocutaneous lesions such as nodular or ulcerative dermatitis, as well as generalized weakness, weight loss, anorexia, and oral or ocular involvement affecting the conjunctiva, cornea, uvea, and retina (Pennisi and Persichetti 2018). The most frequently observed clinicopathological abnormalities are non-regenerative anemia, hyperproteinemia characterized by hyperglobulinemia and hypoalbuminemia, and proteinuria (Pennisi and Persichetti 2018).

Accurate diagnosis of FeL relies on the use of both direct and indirect detection methods in clinical and epidemiological settings. Direct diagnostic techniques include polymerase chain reaction (PCR), cytology, histology, immunohistochemistry, and parasite culture. Indirect serological methods comprise the immunofluorescent antibody test (IFAT), direct agglutination test (DAT), enzyme-linked immunosorbent assay (ELISA), and western blot (WB). However, these diagnostic tools alone provide limited insight and lack sufficient clinical relevance. Therefore, combining methods of different natures, particularly serological and molecular approaches, represents a more reliable strategy for the detection of FeL. This integrated approach improves diagnostic accuracy by compensating for the inherent limitations of each individual technique, highlighting the importance of an integrated diagnostic approach (Alcover et al. 2021).

In dogs, CD4⁺ Th1 lymphocytes produce *Leishmania*-specific interferon-γ (IFN-γ), which activates macrophages into effector cells capable of controlling parasite replication and disease progression. In contrast, dogs with clinical leishmaniosis and poor outcomes generally exhibit a reduced or absent cell-mediated immune response (Solano-Gallego et al. 2011). In cats, information regarding the immune response remains limited compared to dogs. However, recent evidence indicates that cats are capable of developing both parasite-specific cell-mediated and humoral adaptive immune responses (Priolo et al. 2019, 2022).

In dogs affected by non-controlled leishmaniosis, higher anti-*Leishmania* antibody levels, as reflected by elevated IFAT titers, are positively correlated with disease severity and clinical scores, a relationship that becomes particularly evident in advanced clinical forms (Proverbio et al. 2014). The excessive production of anti-*Leishmania* antibodies, together with the presence of circulating parasite antigens, promotes the formation of circulating immune complexes (CIC) composed of aggregated *Leishmania* proteins, specific IgG and IgM antibodies and, to a lesser extent, Complement System components (Parody et al. 2019; Gizzarelli et al. 2020). These CIC can deposit in various tissues and organs, leading to inflammatory and immune-mediated lesions such as vasculitis, uveitis, dermatitis, and, most notably, glomerulonephritis and renal failure. In this sense, detection of CIC in cases of CanL could be an alternative disease biomarker not only for diagnosis but also for tracking disease progression and potentially for monitoring the success of treatment in dogs (Parody et al. 2019; Osuna et al. 2022).

*Leishmania*-specific diagnostic test to measure CIC in cats is currently unavailable in clinical practice. Our study addresses this critical gap by focusing on the validation in feline model of this specific method developed in 2019 for canine *Leishmania* model (Parody et al. 2019).

We suggest that CIC could be a valuable biomarker for diagnosis, and disease progression monitoring in cats, as previously has been demonstrated in dogs (Sarquis et al. 2024). Our study contributes to enhancing diagnostic approaches for FeL and underscores the potential of CIC as a complementary tool in veterinary practice. As we move forward, larger studies will be essential to confirm these findings and establish a definitive cut-off for clinical application.

This report presents the first documented case of detection of CIC in a naturally infected cat with *L. infantum*, along with a short-term follow-up associated with anti-*Leishmania* treatment.

## Case presentation

### Case history

An 8-year-old female spayed European Shorthair cat was referred to Ophthalmologist Service Zaragoza Veterinary Faculty (Spain). The cat was appropriately vaccinated and dewormed. Four months earlier, the cat had been diagnosed with uveitis in the right eye, which initially responded to symptomatic treatment with topical glucocorticoids, cycloplegics, and antibiotics, but relapsed upon discontinuation of therapy. Diagnostic imaging, including thoracic radiography and abdominal ultrasonography, revealed no detectable abnormalities. Complementary serological tests had previously been performed at URANOLAB^®^ to detect antibodies against *Toxoplasma gondii* and feline immunodeficiency virus (FIV), as well as antigens of *Cryptosporidium* spp. (feces) and feline leukemia virus (FeLV) (serum), with negative results for all tests.

At the initial physical examination, the cat was alert, with a body weight of 5.2 kg and a body condition score of 3/5. The animal was normothermic, adequately hydrated, and exhibited pink mucous membranes. Abdominal palpation revealed a mildly distended but non-painful abdomen, without evidence of organomegaly or palpable masses. Cardiac auscultation and respiratory sounds were within normal limits. Mild enlargement of the submandibular lymph node ipsilateral to the eye with uveitis was detected, and the mucosal surfaces (vaginal, ocular, nasal, and rectal) appeared normal. Apart from ocular lesions, the general examination was unremarkable.

**Fig. 1:**
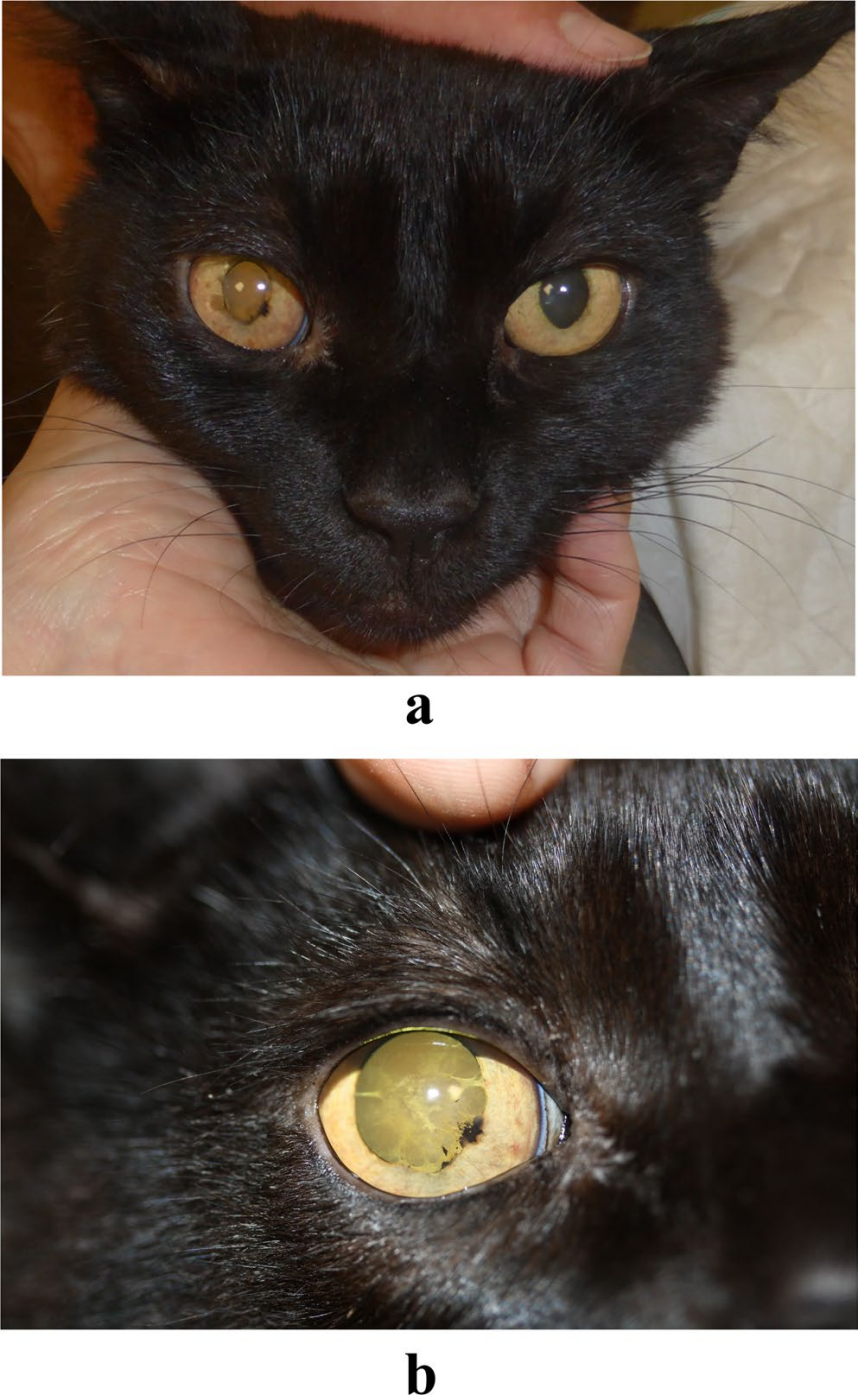
A 8-year-old female spayed European Shorthair cat with unilateral exudative uveitis in the right eye at first presentation (**A**). Appearance of the right eye after one month of treatment. Iris inflammation has decreased markedly; a ventral synechia persists. An incipient cataract secondary to the initial uveitis is observed (**B**)

Ophthalmic examination showed a normal menace response and palpebral reflex in both eyes. In the right eye (OD), the direct pupillary light reflex was absent, while the consensual response was present. In the left eye (OS), the direct reflex was present, but the consensual response was absent. Intraocular pressure measured by rebound tonometry (iCare IC100) was 15 mmHg in OD and 18 mmHg in OS. Slit-lamp biomicroscopy revealed mild aqueous flare (Tyndall effect), iritis, and a miotic pupil with posterior synechiae in OD, whereas OS appeared normal. Following topical instillation of tropicamide (Tropicamide 10 mg/ml, ophthalmic solution), fundus examination of OD was not possible due to the presence of an incipient cataract, while fundoscopic evaluation of OS was unremarkable. Based on these findings, a diagnosis of unilateral exudative uveitis was established (Fig. 1a).

Due to the lack of sustained response to previous medical treatment, the cat was sedated for further diagnostic procedures, including ocular and abdominal ultrasonography and aqueous humor sampling for cytological examination. Sedation was achieved using dexmedetomidine (0.8 µg/kg) and butorphanol (0.3 mg/kg). Ocular ultrasonography revealed a focal area of tissue proliferation within the medial region of the ciliary body of the right eye, consistent with a nodular lesion measuring approximately 0.45 cm in diameter.

For aqueous humor collection, the conjunctival sac was disinfected with diluted povidone–iodine (1:50), and aqueocentesis was performed using a 30 G needle inserted through the sclero-corneal limbus to withdraw 0.2 ml of aqueous humor, which was replaced with balanced salt solution (BSS). Cytological examination of the aqueous humor revealed the presence of small lymphocytes with normal morphology, findings inconsistent with lymphoma. The sample was then analyzed by agarose gel electrophoresis (AGE) and subjected to further serological and molecular investigations, including quantitative PCR (qPCR) for *Leishmania* spp. DNA and serological detection of specific anti–*L. infantum* IgG antibodies. Additionally, PCR testing for feline coronavirus in the aqueous humor sample yielded a negative result.

## Clinicopathological findings

**Fig. 2:**
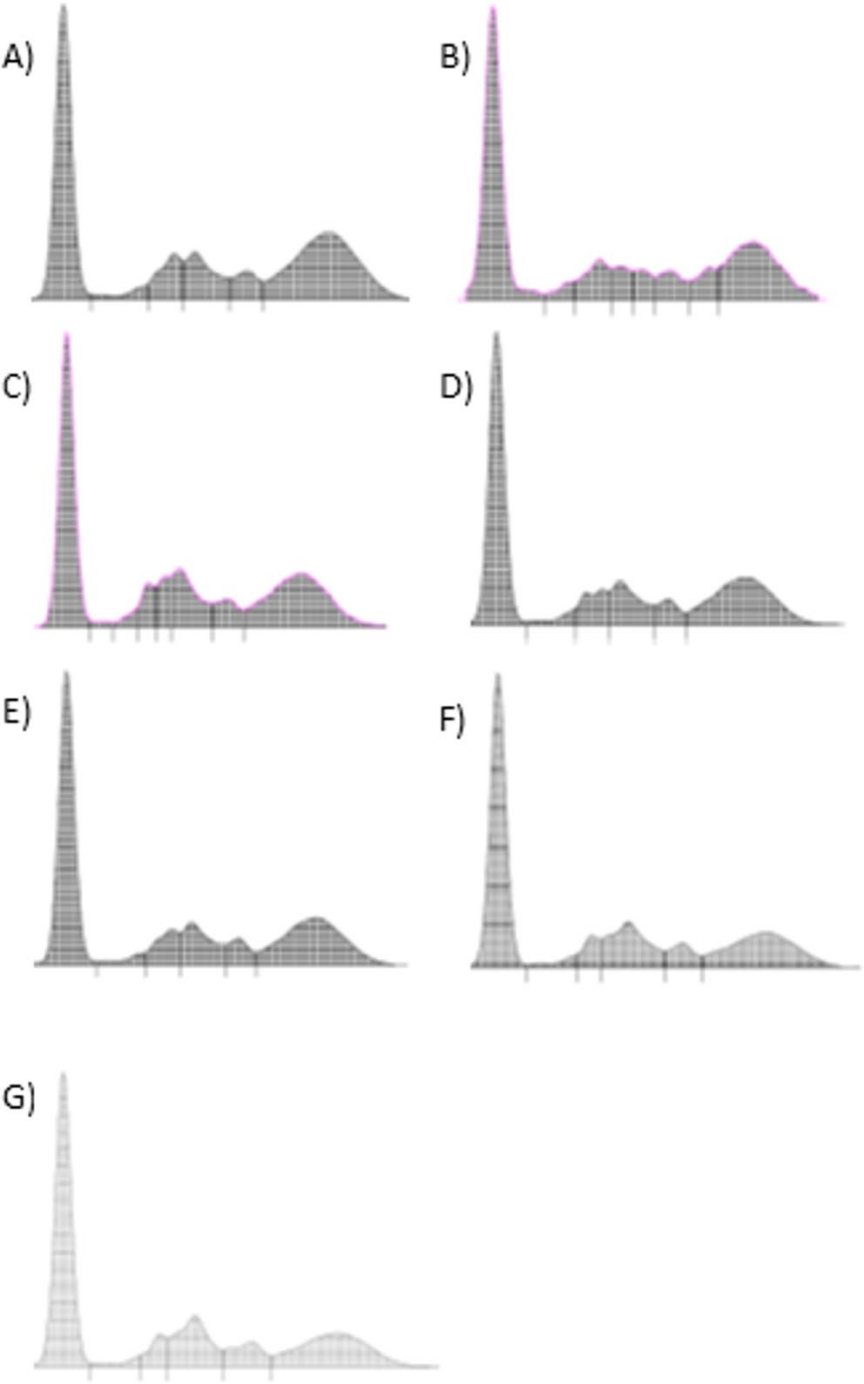
Agarose gel electrophoresis of serum proteins (**A**) and aqueous humor (**B**) before starting allopurinol treatment and during the follow-up in serum samples (**C**, **D**, **E**, **F**). **C** Two-weeks follow-up. **D** Four-weeks follow-up. **E** Nine-weeks follow-up. **F** Fifteen-weeks follow-up. **G** Twenty-three weeks follow-up

A complete blood count (CBC) (Procyte Dx^®^, Idexx, Westbrook, Maine, USA) and serum biochemistry panel (Catalyst One^®^ Chemistry Analyzer, Idexx, Westbrook, Maine, USA) were also conducted, including glucose, creatinine, blood urea nitrogen, alanine aminotransferase, and alkaline phosphatase. Urine analysis was performed including urine specific gravity, urine sediment analysis and urine protein to creatinine ratio. Serum protein electrophoresis was performed by AGE (Hydragel Kit 1–2, Sebia, Issy-les-Moulineaux, France). Serum was electrophoresed for 21 min at 92 V and stained with diluted amidoschwarz dye at pH 2 (4 g/L amidoschwarz dye and 6.7% ethylene glycol). The AGE procedure was conducted according to the manufacturer’s instructions. The electrophoretic curve for each sample was displayed and read with a GELSCAN TM densitometry system (Sebia, Issy-les-Moulineaux, France). The only main clinicopathological finding was the presence of a very mild hyperglobulinemia (Table 1) and the AGE indicated a polyclonal gammopathy (Fig. 2a) and the same electrophoretic pattern was detected in the aqueous humor sample (Fig. 2b).

**Table 1:** Haematological, biochemical parameters and diagnostic confirmatory techniques serology determined in the Leishmaniotic cat at the first veterinary examination before treatment and during the follow-up

*Visits*	First visit	Second visit (Two weeks later)		Third visit (Four weeks later)	Fourth visit (Nine weeks later)		Fifth visit (Fifteen weeks later)		Sixth visit (Twenty-three weeks later)	Seventh visit (Twenty-seven weeks later)	Reference Range
*Haematology*											
WBC (K/µl)	5.25	3.82		3.23	4.25		5.59		3.36	NA	2.87–17.02
Neutrophils (K/µL)	2.43	1.42		1.19	0.99		2.98		0.80	NA	2.30-10.29
Lymphocytes (K/µL)	2.43	1.98		1.83	2.83		2.09		2.23	NA	0.92–6.88
Monocytes (K/µL)	0.37	0.40		0.21	0.42		0.42		0.33	NA	0.05–0.67
Eosinophils (K/µL)	0.00	0.01		0.00	0.01		0.02		0.00	NA	0.17–1.57
Basophils (K/µL)	0.02	0.01		0.00	0.00		0.08		0.00	NA	0.01–0.26
RBC (M/µL)	11.22	11.01		10.01	11.01		10.18		9.96	NA	6.54–11.20
Haematocrit (%)	51.0	50.0		46.6	49.9		49.6		48.7	NA	30.30–52.30
Haemoglobin (g/dL)	16.4	15.2		13.8	15.2		15.0		14.8	NA	9.80–16.20
Reticulocytes (K/µL)	18.0	12.1		7.0	14.3		8.1		9.0	NA	3.00–50.00
MCV (fL)	45.5	45.4		46.6	45.3		48.7		48.9	NA	35.90–53.10
MCH (pg)	14.6	13.8		13.8	13.8		14.7		14.9	NA	11.80–17.30
MCHC (g/dL)	32.2	30.4		29.6	30.5		30.2		30.4	NA	28.10–35.80
Plateles (K/µL)	458	175		301	273		201		306	NA	151–600
*Blood Chemistry*											
Alanine aminotransferase (U/L)	28	30		97	63		20		12	NA	12–130
Alkaline phosphatase (U/L)	20	25		19	28		35		38	NA	14–111
Glucose (mg/dL)	100	90		97	121		100		93	NA	74–159
Creatinine (mg/dL)	1.2	1.1		0.7	0.8		0.8		1.0	NA	0.8–2.4
Blood Urea Nitrogen (mg/dL)	19	22		31	30		25		22	NA	16–36
*Electrophoretograms of serum proteins (AGE)*											
Total protein (g/dL)	8.1	8.1		8.0	7.9		7.8		8.2	NA	5.7–7.9
Albumin (g/dL)	3.3	3.6		3.6	3.3		3.6		3.8	NA	2.1-4.0
Alpha 1 globulins (g/dL)	0.2	0.2		0.2	0.2		0.2		0.2	NA	0.1–1.10
Alpha 2 globulins (g/dL)	0.7	0.6		0.6	0.4		0.4		0.4	NA	0.4–0.9
Beta globulins (g/dL)	1.2	1.4		1.3	1.8		1.8		1.9	NA	0.3–0.7
Gamma globulins (g/dL)	2.7	2.3		2.3	2.2		1.8		1.9	NA	0.9–1.90
A/G	0.69	0.80		0.81	0.72		0.86		0.87	NA	1.30–2.20
											0.45–1.30
*Urine analysis*											
Urine specific gravity	> 1,050	NA		NA	NA		NA		NA	> 1,050	1,030 − 1,060
Urine protein to creatinine ratio	< 0.18	NA		NA	NA		NA		NA	< 0.20	< 0.4
Sediment	-	NA		NA	NA		NA		NA	- (absence of xanthinas)	
*Leishmania infantum *ELISA Serology											
Serum sample One dilution (1/100) (OD units)	2.246	2.227		2.176	1.316		1.248		1.175	NA	≥ 0.130
Two-fold serial dilution (OD unit)	675	436		330	168		167		118	NA	
										NA	
Aqueous humor										NA	
One dilution (1/100) (OD units)	0.660	NA		NA	NA		NA		NA	NA	Cut off ≥ 0.130
Two-fold serial dilution (OD unit)	66	NA		NA	NA		NA		NA	NA	
*Leishmania infantum* *Western Blot Serology*											Cut-off 14 and/or 16 kDa
Serum sample	14,16, 18, 24, 26, 28, 30, 36, 42, 50, 70	NA		NA	NA		NA		NA	NA	
*Leishmania* *feline)Leishmania infantum* *rapid test* (Uranotest^®^											
Serum sample	+	+		+	+		+		+	NA	
Aqueous humor	+	NA		NA	NA		NA		NA	NA	
* Molecular (qPCR)*											
EDTA-Blood	25	NA		NA	NA		NA			NA	Cq < 40
Aqueous humor	36	NA		NA	NA		NA			NA	
*Leishmania infantum* *isolation*											
Submandibular lymph node	+	NA		NA	NA		NA			NA	

*A/G* albumin: globulin ratio, *MCH* mean corpuscular haemoglobin, *MCHC* mean corpuscular haemoglobin concentration, *MCV* mean corpuscular volume, *NA* not available, OD optical density, *RBC* red blood count, *RDW* red blood cell distribution, *WBC* white blood count, *+* positive, - negative

A panel of confirmatory tests for *L. infantum* infection was performed, including parasite culture and isolation, serological methods and qPCR.

### Detection of *L. infantum* antibodies by serological techniques

**Fig. 3:**
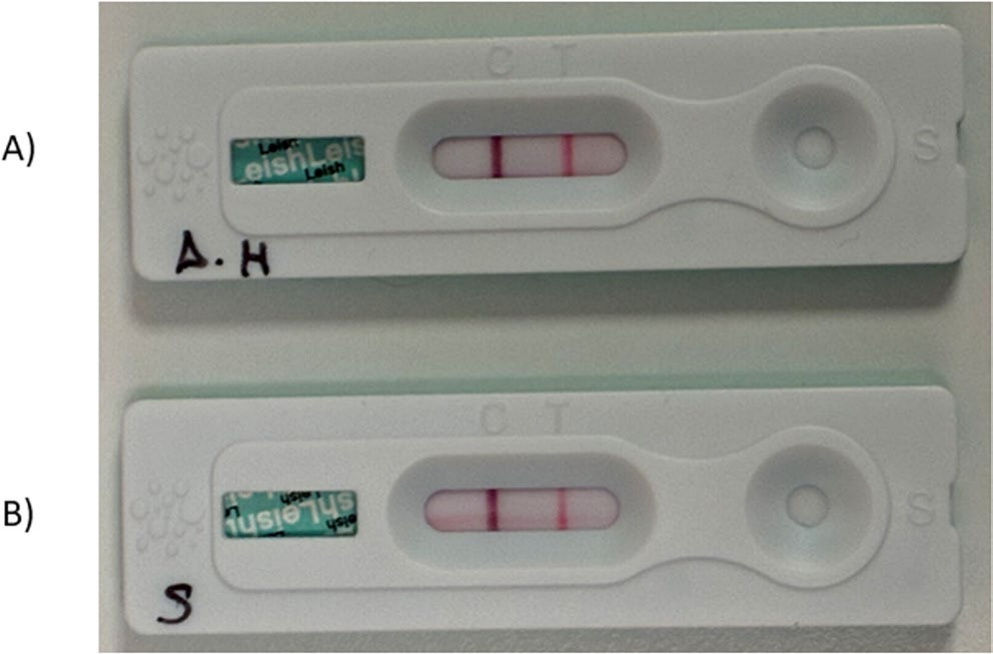
Uranotest^®^ *Leishmania* Feline immunochromatographic rapid tests were performed on aqueous humor (**A**) and serum (**B**) samples before starting allopurinol treatment. Both tests (A) and (B) were interpreted as positive results, with two lines appearing in the result window. Regardless of which line appears first, the result is considered positive

The rapid test (Uranotest^®^ *Leishmania* felino, Uranovet, Spain) was performed following the instructions of the manufacturer. All tests were stored at room temperature and were performed as described in the instructions supplied with the test kit. A positive result was obtained for this test in serum sample (Fig. 3a) and aqueous humor (Fig. 3b).

An ELISA for the detection of antibodies *against L. infantum* was performed on all serum samples following the protocol described previously (Alcover et al. 2021). As a positive control (calibrator), each plate included serum from a cat from Spain diagnosed with FeL, and as a negative control, serum from a healthy, non-infected cat. All samples and controls were tested in duplicate, with the cutoff set at 0.130 optical density units and the results above this value were considered positive. Two-fold serial dilutions were then prepared, beginning at 1:100 and continuing through ten subsequent dilutions on the same ELISA plate. The dilution at which the OD approached 1.00 was used for the final result, calculated using the following formula: (sample OD/calibrator OD) × dilution factor. High anti-*Leishmania* antibodies were detected in serum, whilst low levels of antibodies were detected in aqueous humor sample (Table 1).

For WB analysis, anti-*Leishmania* antibodies were detected using a whole *L. infantum* promastigote antigen (MHOM/FR/78/LEM75 zymodeme MON-1) (Alcover et al. 2021). A serum sample was considered WB-positive if immunoreactivity against the 14 kDa and/or 16 kDa low-molecular-weight polypeptide fractions of the *L. infantum* antigen were observed. WB analysis demonstrated immunoreactivity against *Leishmania* antigen fractions of 14,16, 18, 24, 26, 28, 30, 36, 42, 50 and 70 kDa in serum sample.

Finally, the presence of *Leishmania* spp. DNA in EDTA-blood and an aqueous humor sample were evaluated by amplification of kinetoplast DNA sequence using a quantitative polymerase chain reaction (qPCR), as previously described (Alcover et al. 2021). Each amplification was performed in triplicate in a 10-µl reaction mixture containing 1× iTaq supermix with Rox (Bio-Rad), 15 pmol of direct primer (5′-CTTTTCTGGTCCTCCGGGTAGG-3′), 15 pmol of reverse primer (5′-CCACCCGGCCCTATTTTACACCAA-3′), 50 pmol of the labeled TaqMan probe (FAM-TTTTCGCAGAACGCCCCTACCCGCTAMRA) and 2.5 µl of sample DNA. Cycling was performed using the ABI Prism 7900 system (Applied Biosystems [Thermo Fisher Scientific], Foster City, CA, USA) at 94 °C/55°C for 40 cycles. A non-template control was used in each run as the qPCR negative control. A tenfold dilution series of DNA from promastigotes (MHOM/ES/04/BCN-61, *L. infantum* zymodema MON-1) was used for calibration (serial dilution from 10^5^ to 10^− 3^ parasites/ml), allowing the plotting of a standard curve. The qPCR was considered positive for *Leishmania* when the quantification cycle (Cq) was < 40 and the amplification was detected in all the replicates. A positive PCR result was obtained from both samples, with a Cq of 25 in the EDTA-blood and a Cq of 35 in the aqueous humor sample.

### *Leishmania infantum* in vitro isolation and cultivation

For parasite isolation, in-house Novy–MacNeal–Nicolle (NNN) medium and commercial Schneider medium, each supplemented with 100 IU/mL penicillin, 100 µg/mL streptomycin, and 10% fetal calf serum (Thermo Fisher scientific), were used. Several drops of aspirated material from the lymph node ipsilateral to the uveitis were introduced into the liquid phase of the NNN medium and then incubated in Schneider medium at 26 ± 1 °C. Cultures were examined microscopically on a daily basis (Giner et al. 2020). Parasite culture became positive after sixteen days of incubation. The *Leishmania* spp. strain isolated (Clinical Immunology Laboratory, University of Zaragoza) was molecularly typed as *L. infantum* (Laboratory of Parasitology, University of Barcelona) and designated ZGZ-CAT1 (Alcover et al. 2021).

### Circulating immune complexes detection

CIC were measured using an adapted *Leishmania*-specific ELISA method described previously (Parody et al. 2019) for use in dogs. A modified precipitation method with polyethylene glycol (PEG; Sigma-Aldrich, St Louis, MO, USA) was used to precipitate and isolate CIC. PEG-precipitated CIC were pelleted by centrifugation, then reconstituted in 0.01 M phosphate-buffered saline (PBS) and stored at − 80 °C for further use. Briefly, microtiter immunoassay plates coated with Soluble *Leishmania* Antigens (SLA) were used to detect *Leishmania*-specific CIC. Microplates were incubated with a 1:100 dilution of PEG-precipitated CIC for 1 h at room temperature (RT). Microplates were then washed and incubated with Peroxidase-conjugated anti-cat IgG (Merck, Darmstadt, Germany) (1:5,000) at RT for 1 h. After washing, microplates were developed with a solution of SIGMAFAST™, peroxidase substrate, chromogenic, tablet (OPD) (Merck, Darmstadt, Germany). After 15 min, the reaction was stopped by adding a solution of 3 MH2SO4, and microplates read at 492 nm in an ELISA microplate spectrophotometer (Bio-Rad). All samples were tested in duplicate, and the mean value was recorded. In order to define the cutoff value, 20 samples from control cats (healthy and negatives in a *Leishmania*-specific ELISA) were tested. The cutoff was determined as the mean OD + 2 Standard Deviation (0.110) of the control group (negative samples); any sample exhibiting absorbance above the cutoff value was considered positive. In this sense, a value of 0.372 was obtained at first presentation.

### Analysis for other infections

Additionally, the animal was tested for co-infections with other infectious disease agents. This included the detection of specific antibodies against *Dirofilaria immitis* (Villanueva-Saz et al. 2021a), T.*gondii* (Villanueva-Saz et al. 2022), *Bartonella henselae* (MegaFLUO^®^ *BARTONELLA henselae)*, and FIV (Uranotest^®^ FeLV-FIV), as well as antigen detection for FeLV (Uranotest^®^ FeLV-FIV) in serum. Molecular tests were also performed in a private laboratory to identify genomic DNA from *Mycoplasma haemofelis*, *Candidatus* Mycoplasma haemominutum, *Bartonella* spp., *Ehrlichia* spp., and *Anaplasma* spp. in a private laboratory. All tests yielded negative results for these pathogens.

### Clinical outcome and follow-up

Following diagnosis, anti-*Leishmania* therapy was initiated with allopurinol (20 mg/kg orally every 24 h, administered indefinitely), in combination with topical ophthalmic treatment consisting of prednisolone 10 mg/ml ophthalmic suspension (one drop every 8 h), ketorolac tromethamine 5 mg/ml ophthalmic solution (one drop every 8 h), tropicamide 10 mg/ml ophthalmic solution (one drop every 12 h), and dorzolamide hydrochloride 20 mg/ml ophthalmic solution (one drop every 12 h). Additionally, follow-up visits were performed, including a CBC, serum biochemistry panel, serum protein electrophoresis, Uranotest^®^ *Leishmania* feline, an in-house ELISA for detecting anti-*Leishmania* antibodies, and an specific method to CIC detection.

The cat was re-evaluated 15 days after initiating anti-*Leishmania* therapy with allopurinol. The owners reported a noticeable improvement in the cat’s activity (Table 1). On general physical examination, no abnormalities were detected. Neuro-ophthalmic findings were comparable to those of the initial evaluation. Tonometry values were within the normal range (15 mmHg in both eyes). Slit-lamp biomicroscopy of the right eye revealed mydriasis, ventral and medial posterior synechiae, and incipient cataract. No clinical signs of active uveitis were observed in the right eye, while all findings in the left eye remained within normal limits. Given the absence of clinical signs of uveitis, the frequency of topical ophthalmic medication administration in the right eye was reduced as follows: prednisolone 10 mg/ml ophthalmic suspension (one drop every 12 h for 15 days), ketorolac tromethamine 5 mg/ml ophthalmic solution (one drop every 12 h), and tropicamide 10 mg/ml ophthalmic solution (one drop every 24 h). A follow-up examination was performed 15 days later (Fig. 1b), at which time the cat remained stable both systemically and ophthalmologically. The clinical signs of uveitis in the right eye had resolved (Fig. 1b), and topical anti-inflammatory therapy was discontinued while maintaining the allopurinol regimen during the follow-up (Table 1).

**Fig. 4:**
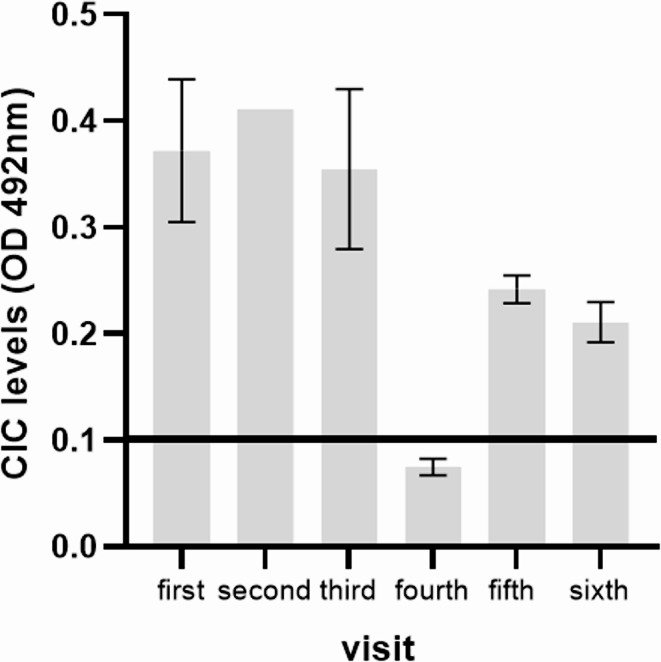
CIC levels before starting anti-*Leishmania* treatment (visit 1) and during the follow-up: visit 2 corresponds two-weeks follow-up. Visit 3 corresponded to four-week follow-up. Visit 4 corresponded to nine-weeks follow-up. Visit 5 corresponded to fifteen-weeks follow-up. Visit 6 corresponded to twenty-three-weeks follow-up

During follow-up evaluations, the cat appeared healthy, and repeated haematological and biochemical tests showed no significant alterations. In addition, there was an initial increase in CIC levels two weeks after starting allopurinol treatment (0.411), but subsequently a marked reduction was observed four weeks later (0.355), with CIC becoming undetectable nine weeks (0.075) after diagnosis. However, CIC levels were also detected in the follow-up during the fifth visit (0.242) and sixth visit (0.211) (Fig. 4).

### Discussion and conclusions

To our knowledge, this is the first study to assess the clinical use of CIC as biomarker for feline leishmaniosis. This method was validated for dogs but must be clinically validated in feline leishmaniosis as a new biomarker not only for a diagnosis but also for tracking disease progression and potentially assessing treatment efficacy.

In dogs, clinical classification of CanL includes an integrating approach encompassing serological status, clinical signs and prognosis for each stage based on LeishVet group guidelines (Solano-Gallego et al. 2011). Recently, CIC level has been evaluated as possible parameter to include in this classification (Sarquis et al. 2024). According to the LeishVet guidelines, dogs can be categorized into five main clinical stages, ranging from noninfected to very severe disease. Non-infected and infected asymptomatic dogs typically exhibit negative serology and CIC levels and remain free of clinical signs, with an excellent overall prognosis. Disease becomes clinically evident in Stage I, where dogs show negative or low antibody titers and low CIC levels. Clinical signs are generally mild at this stage and may include peripheral lymphadenomegaly or papular dermatitis, with a prognosis that remains favorable to guarded. Progression to Stage II is characterized by low to high serological antibody levels and low to medium CIC levels. Dogs in this stage present more noticeable clinical manifestations in addition to those described in Stage I. These may include diffuse or symmetrical dermatological lesions such as exfoliative dermatitis or onychogryphosis, ulcerations at mucocutaneous junctions or bony prominences, as well as systemic signs like anorexia, weight loss, fever, and occasional epistaxis. Prognosis in this stage varies from guarded to good, depending on the response to treatment and the presence of complications. The next level, Stage III reflects a severe disease condition, with medium to high antibody titers and elevated CIC levels. Alongside the signs observed in earlier stages, clinical manifestations associated with immune complex deposition become more evident, particularly vasculitis, arthritis, uveitis, and glomerulonephritis. Finally, Stage IV represents very severe disease, also associated with medium to high antibody and elevated CIC levels. In addition to the signs of Stage III, affected animals commonly develop life-threatening complications such as nephrotic syndrome, pulmonary thromboembolism, or end-stage renal disease. At this advanced stage, prognosis is poor.

Due to the lack of information related to CIC and FeL, the main clinical finding detected in this animal was the presence of a uveitis, being this clinical finding associated in dogs with CanL with a Stage III with immunocomplex deposition. In cats with *L. infantum* infection, uveitis may reflect more severe clinical involvement and may be associated with higher CIC levels. Fifteen days after starting anti-*Leishmania* treatment composed by allopurinol therapy, no clinical signs of active uveitis were observed, although the levels of CIC increased compared to the concentration at diagnosis time.

In dogs, anti-*Leishmania infantum* antibodies can be detected in biological fluids other than serum, including oral transudate (Baxarias et al. 2022), urine (Solano-Gallego et al. 2003), cerebrospinal fluid (Oliveira et al. 2017), peritoneal effusion (Villanueva-Saz et al. 2020), and aqueous humor (El Goulli et al. 2023). In cats, the presence of antibodies against *L. infantum* in aqueous humor has also been reported recently (Schäfer et al. 2023). Moreover, in our case confirmatory molecular testing detected *Leishmania* DNA in the ocular sample. Accordingly, identifying the parasite by both molecular and serological methods in the affected organ strengthens a cause-effect relationship. Aqueous humor protein electrophoresis revealed a pattern similar to that of serum protein analysis, a finding likely related to uveitis pathogenesis due to increased permeability of the blood-aqueous barrier.

In dogs with uveitis, anti-*Leishmania* antibodies have been detected at higher levels in ocular samples than in serum; in some cases, serum antibodies are undetectable while aqueous humor is seropositive, suggesting local antibody production (El Goulli et al. 2023). In cats, however, published data are limited to this report and one previous clinical case (Schäfer et al. 2023). In both feline cases, antibody levels in aqueous humor were lower than in paired serum samples. Taken together with the similar electrophoretic patterns observed in serum and aqueous humor, these findings are more consistent with increased blood–aqueous barrier permeability than with local intraocular antibody production driven by parasite presence within the ocular lesion.

This cat presented with uveitis as the main ophthalmic finding, together with mild enlargement of the submandibular lymph node, compatible with FeL. In general, most reported FeL cases show cutaneous or mucocutaneous lesions with lymphadenomegaly, sometimes accompanied by nonspecific clinical signs (Pennisi et al. 2015; Pennisi and Persichetti 2018). Some cats exhibit purely dermatologic involvement (Rüfenacht et al. 2005), whereas others lack skin lesions at the time of examination (Migliazzo et al. 2015), as in the present case.

Regarding clinicopathologic abnormalities in FeL, a variable, normocytic, normochromic, nonregenerative anemia is the most frequently reported hematologic change (Pennisi et al. 2015). By contrast, the complete blood count in this case was unremarkable. Hyperproteinemia due to hypergammaglobulinemia was detected, which is a common laboratory finding in dogs (Paltrinieri et al. 2016), cats (Pennisi and Persichetti 2018) and ferrets (Villanueva-Saz et al. 2021b) with clinical leishmaniosis.

After *Leishmania* infection, when antigen levels are high and antibodies are abundant, antigen-antibody complexes form in the bloodstream resulting in the called CIC. These immunocomplexes are normally cleared efficiently by the mononuclear phagocytic system. But in clinically affected dogs, the continuous production of anti-*Leishmania* antibodies together with a high parasite burden leads to persistent formation of CIC and their subsequent deposition in tissues, once the clearance capacity of the mononuclear phagocytic system is exceeded. In this cat, positive correlation between clinical score and CIC levels were found according to previous studies in dogs that suggest a decrease in clinical score will be accompanied by a drop in CIC levels (Sarquis et al. 2024). CIC levels increased again at 15 weeks after diagnosis (fifth visit), although to a lesser extent than at the time of diagnosis, and then decreased by 23 weeks after diagnosis (sixth visit). Cats infected with *L. infantum* may develop a heterogeneous and sometimes fluctuating immune response, in which antibody titers do not always mirror disease activity. In such situations, serology alone may be insufficient to characterize ongoing antigenic stimulation, particularly when humoral responses are modest, delayed, or variable. Because CIC formation depends on the simultaneous presence of parasite antigen and specific antibodies, CIC quantification may offer complementary information by integrating elements of antigen load and host response. This could be especially relevant in cats with an unstable immune profile, in whom antibodies are detectable but not strongly predictive of clinical status.

In CanL CIC levels dropped after treatment in good responder dogs not so in bad responders, suggesting that measuring CIC levels before and after treatment could serve to assess treatment effectiveness (Sarquis et al. 2024). In this animal CIC levels dropped after allopurinol treatment suggesting a good responder cat in combination of a decrease of anti-*Leishmania* antibodies levels detected by ELISA and a normalization of serum protein electrophoresis profile and the absence of clinical and laboratory abnormalities. Further, it has been published that measuring CIC levels could be useful in dogs that do not have an overall picture of generalized CanL yet show clinical signs of deposition of CIC (Cacheiro-Llaguno et al. 2021).

Several therapeutic approaches involving different treatment regimens were reported in the management of feline leishmaniosis. Specifically, in a recent revision of clinical management of feline leishmaniosis caused by *L. infantum* (Garcia-Torres et al. 2022), allopurinol was the most frequently administered drug, followed by meglumine antimoniate, while miltefosine was used in only one case. The most commonly used regimen was for allopurinol was 10 mg/kg twice daily (PO) for a minimum of six months, for meglumine antimoniate at 50 mg/kg once daily subcutaneously for 30 days, and for miltefosine at 2 mg/kg once daily *per os* for 28 days in the single cat that received this drug. Allopurinol was predominantly used as monotherapy, whereas combination protocols including either meglumine antimoniate or miltefosine were each reported in only one case. It is important to note that propylene glycol is one of the excipients in the oral miltefosine formulation approved for the treatment of CanL (Milteforan™). Since propylene glycol can induce Heinz body formation and reduce the lifespan of feline red blood cells, this may explain the lack of reported use of miltefosine in cats (Pennisi and Persichetti 2018). In some sick cats, meglumine antimoniate was also administered as monotherapy. Adverse effects associated with allopurinol treatment have been reported, including increased liver enzyme activity, renal complications, and toxidermia (Pennisi et al. 2013). In general, anti-*Leishmania* treatment for feline leishmaniosis improves clinical signs, prolongs survival, and controls the disease; however, in some animals it results in only a partial cure, and there remains a risk of relapse (Mestrinho et al. 2022; Napoli et al. 2022; Matralis et al. 2023; Tiozzo et al. 2023).

This report describes a single naturally infected cat; therefore, the observed CIC kinetics and their apparent relationship with clinical improvement and antibody dynamics cannot be generalised to FeL. Moreover, the CIC assay, adapted from canine methodology, requires validation in larger feline cohorts including definition of cut-off values and assessment of analytical performance. Prospective studies with standardised protocols and longer follow-up are needed to clarify the diagnostic and monitoring value of CIC in cats.

In our cat, administration of allopurinol resulted in an improvement of the animal’s condition, together with a decrease in anti-*Leishmania* antibody levels and CIC. The decrease in both parameters correlates with clinical improvement and with the efficacy of the anti-*Leishmania* treatment. To the best of our knowledge, this is the first time the relationship between treatment efficacy and the reduction of CIC has been demonstrated in this cat with leishmaniosis supporting their potential role as a biomarker not only in dogs but also in cats infected with *L. infantum*. Further studies are needed to verify the optimal use of this new promising biomarker in monitoring FeL, particularly in animals with lesions caused by CIC deposition.

## Data Availability

Not applicable.
